# Analysis of Atmospheric CO_2_ and CO at Akedala Atmospheric Background Observation Station, a Regional Station in Northwestern China

**DOI:** 10.3390/ijerph19116948

**Published:** 2022-06-06

**Authors:** Zhujun Zhao, Qing He, Zhongqi Lu, Quanwei Zhao, Jianlin Wang

**Affiliations:** 1 Institute of Desert Meteorology, China Meteorological Administration, Urumqi 830002, China; putaojing1995@163.com (Z.Z.); lzq101016@163.com (Z.L.); 2 College of Resources & Environmental Sciences, Xinjiang University, Urumqi 830017, China; 3 Field Scientific Experiment Base of Akedala Atmospheric Background Station, China Meteorological Administration, Altay 836500, China; wjl20220120@126.com; 4 Xinjiang Meteorological Service Center, Urumqi 830002, China; zqw311403020128@163.com; 5 Akedala Atmospheric Background Station, Altay 836500, China

**Keywords:** CO_2_ and CO mole fraction, hybrid single-particle Lagrangian integrated trajectory, potential source region

## Abstract

Air samples were collected by flasks and analyzed via a Picarro G2401 gas analyzer for carbon dioxide (CO_2_) and carbon monoxide (CO) at the Akedala Atmospheric Background Station in Xinjiang, China, from September 2009 to December 2019, to analyze the changes in the characteristics of atmospheric CO_2_ and CO and determine the sources. The results show that the annual average CO_2_ concentration showed an increasing trend (growth rate: 1.90 ppm year^−1^), ranging from 389.80 to 410.43 ppm, and the annual average CO concentration also showed an increasing trend (growth rate: 1.78 ppb year^−1^), ranging from 136.30 to 189.82 ppb. The CO_2_ concentration and growth rate were the highest in winter, followed by autumn, spring, and summer. The CO concentration and growth rate were also the highest in winter due to anthropogenic emissions, ecosystem effects, and diffusion conditions. The main trajectories of CO_2_ and CO determined by the Hybrid Single-Particle Lagrangian Integrated Trajectory (HYSPLIT) model were parallel to the Irtysh River valley and then passed through the Old Wind Pass. Furthermore, the main source regions of CO_2_ and CO at the Akedala Station were eastern Kazakhstan, southern Russia, western Mongolia, and the Xinjiang Tianshan North Slope Economic Zone of China. This study reflects the characteristics of long-term changes in CO_2_ and CO concentrations at the Akedala station and provides fundamental data for the studies on environmental changes and climate change in Central Asia.

## 1. Introduction

Global warming poses increasing climate risks for Central and East Asia [1]. Several studies have shown that the anthropogenic emission of greenhouse gases such as carbon dioxide (CO_2_) contributes greatly to global warming [1]. The signing of the *United Nations Convention on Climate Change* in 1992, the proposed temperature control of 1.5 °C in the Paris Agreement in 2015, and the goal of “carbon peaking and carbon neutrality” proposed by China in 2021, all indicate that human beings are aware of the negative impacts of greenhouse gas emissions.

Carbon dioxide is a long-lived anthropogenic greenhouse gas. It is the primary cause of the increase in radiative forcing over the last decade [2,3]. The global average CO_2_ concentration was 413.2 ± 0.2 ppm in 2020, increasing by 2.40 ppm year^−1^ in the past ten years. Due to the COVID-19 epidemic, the growth rate of CO_2_ concentration declined in 2020. This proves once again that anthropogenic emissions have a significant impact on atmospheric greenhouse gas concentrations [4]. Carbon monoxide (CO) is not a conventional greenhouse gas. However, as an important reactive gas in the atmosphere, it can react with hydroxyl radicals (OH) to indirectly affect the concentrations of atmospheric greenhouse gases, such as methane (CH_4_), halogenated hydrocarbons, and tropospheric ozone (O_3_) [5,6,7,8]. CO mainly comes from the processes of fossil fuel and biomass combustion and oxidation reaction of methane and other hydrocarbons in the atmosphere [9]. 

China is currently one of the major CO_2_ emitters [10,11]. The widespread use of fossil fuels, combined with low combustion efficiency, has resulted in an increase in atmospheric CO concentrations and air pollution [12,13]. To continuously monitor atmospheric changes, atmospheric background stations have been built in different regions of China according to the World Meteorological Organization/Global Atmospheric Watch (WMO/GAW) programme. The data collected by these stations could help clarify changes in greenhouse gas concentrations and sources. Among the stations, the Shangdianzi National Atmospheric Background Station in Beijing is responsible for monitoring the atmospheric environment of the North China Plain, the Longfengshan Station in Heilongjiang province is responsible for monitoring the atmospheric environment of the Northeast Plain, and the Waliguan Global Atmospheric Background Station located on the Qinghai-Tibet Plateau is responsible for monitoring the atmospheric environment of the Eurasian continent [14,15,16,17]. Since greenhouse gas concentrations vary in different regions and seasons, the analysis of atmospheric greenhouse gas concentrations at different background stations is of great significance for monitoring pollutants on a regional scale, predicting meteorological disasters, and climate change research. Studies have shown that CO_2_ and CO concentrations vary considerably in the eastern monsoon region of China. The CO_2_ concentration at Shangdianzi station is higher than that at Waliguan station [18], and the CO concentration at Waliguan, Longfengshan, and Shangri-La stations has decreased in recent years [19,20]. However, there are currently few studies on the variation of greenhouse gas concentrations in the non-monsoon region of China. Studies have shown that the current level of carbon sinks in China is underestimated [21]. Therefore, it is crucial to analyze the long-term changes in atmospheric greenhouse gas concentrations in northwest China. 

Changes in greenhouse gas concentrations are closely related to anthropogenic activities and climate change [22]. Xinjiang is located in northwest China. The climate in Xinjiang is quite different from that of the eastern monsoon region, which may lead to different changes in CO_2_ and CO background concentrations. At present, there are few studies on the long-term changes in CO_2_ and CO concentrations in northern Xinjiang. Although the Waliguan Atmospheric Background Station is also located in northwest China, it has a higher altitude and a different climate from northern Xinjiang. Moreover, Xinjiang is located in Central Asia. Background concentrations of CO_2_ and CO in Central Asia have not been updated by the World Greenhouse Gas Data Centre for many years.

In this study, the eleven-year (2009–2019) meteorological data and CO_2_ and CO concentration data collected by the Akedala station were subjected to linear trend analysis, correlation analysis, and backward trajectory cluster analysis to investigate the characteristics of CO_2_ and CO concentration changes that affect climate change in northwest China. This study will provide data for accurate carbon accounting and provide a reference for the formulation of regional environmental policies and strategies to control greenhouse gas emissions.

## 2. Materials and Methods 

### 2.1. Study Location and Data

**Figure 1:**
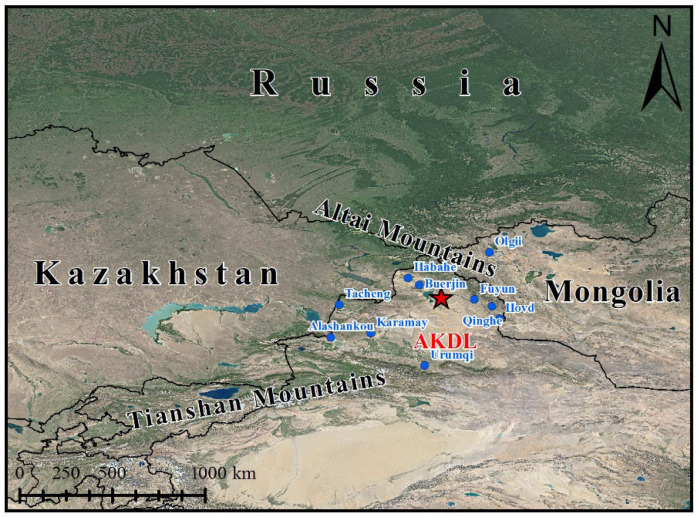
Location of Akedala station. Note: The trapezoidal symbol indicates the sampling site.

**Figure 2:**
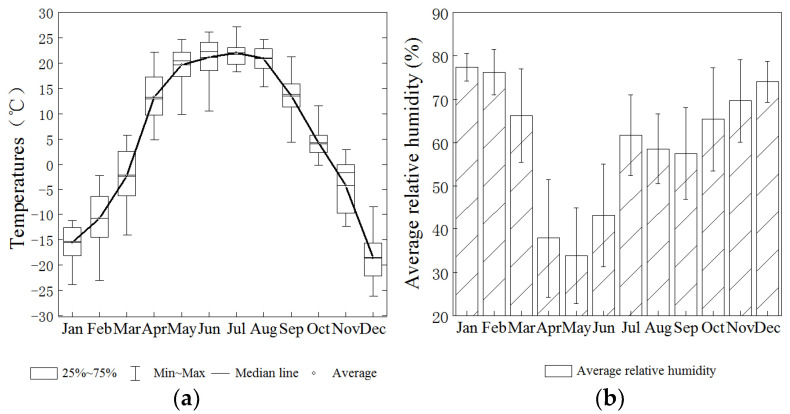
Monthly temperature (**a**) and humidity (**b**) at Akedala station.

Akedala station (87.93°, 47.10° N; 563.3 m a.s.l.), located in the Altay region, Xinjiang, China, was constructed in strict accordance with the Construction Standards of the National Atmospheric Background Station of the China Meteorological Administration (CMA). It is far from cities, and there are no high-intensity anthropogenic emission sources within 50 km (Figure 1) [23]. This region has a typical temperate continental climate (Figure 2), and desert grassland is the dominant landscape [24]. Influenced by the westerly circulation, Akedala station is under the influence of westerly and northwesterly winds throughout the year, except in winter (easterly winds) [25]. The area where the Akedala station is located is mostly influenced by the air masses from Central Asia. Therefore, the observation data at Akedala station could reflect the atmospheric characteristics of northwest China and Central Asia [26,27].

Data on atmospheric CO_2_ and CO concentrations collected by the Akedala station between September 2009 and December 2019 were used for the analysis. Air samples were collected once a week (on Tuesday) using a portable sampler and flasks. The sampling port was placed on a 50 m tower at Akedala station with no shade around it. Sampling was delayed when there was wind (wind speed less than 2 m/s), snow, fog, and rain. To ensure a high mixing degree of the collected air samples and to avoid the influence of human activities and updrafts, the sampling time was set at 14:00 (BST). Before sampling, the flasks were connected in series, inflated with local air for over 10 min, and then pressurized to 1.2–1.5 atm. The air samples collected were analyzed by the Greenhouse Gas Laboratory of the China Meteorological Administration. The technical parameters met the GWO/GAW quality requirements and also fulfilled the requirements of background atmospheric composition analysis [28]. The CO_2_ and CO in air samples were measured using an optical cavity decay spectroscopy analyzer (Model G2401, PICARRO Inc., Santa Clara, CA, USA), and the accuracy of CO_2_ and CO was less than 20 and 1.5 ppb, respectively. Each air sample was analyzed for 5 min, and the last 2 min of the data were averaged to calculate the CO_2_ and CO mole fractions. Some of the observed data, which were potentially affected by improper operations and instability of the instrument during sampling (as judged from target standard gas based on the threshold values of 0.2 ppm and 3 ppb for CO_2_ and CO, respectively), were flagged and excluded.

The observation data of the Waliguan Global Atmospheric Background Station (WLG; 100.54° E, 36.17° N; 3816 m a.s.l.) and the Mauna Loa Global Atmospheric Background Station (MLO; 155.8° W, 19.53° N; 3397 m a.s.l.) in the USA were obtained from the WMO World Greenhouse Gas Data Centre (https://gaw.kishou.go.jp/, accessed on 19 December 2021). The contemporaneous meteorological data for backward trajectory analysis were obtained from the Global Data Assimilation System (GDAS) (6 h interval, horizontal resolution 0.5° × 0.5°) provided by the National Centers for Environmental Prediction (NCEP). Meteorological elements included temperature, pressure, relative humidity, vertical and horizontal wind speeds, and water vapor pressure.

### 2.2. Hybrid Single-Particle Lagrangian Integrated Trajectory (HYSPLIT) Model

The Hybrid Single-Particle Lagrangian Integrated Trajectory (HYSPLIT) model, including a variety of meteorological elements, physical processes, and deposition patterns, was used in the study [29,30,31]. In addition, backward trajectory analysis was carried out based on GDAS data using TrajStat plug-in (http://www.meteothink.org/, accessed on 19 December 2021). The height was 1000 m (higher than the boundary layer at Akedala station), the duration was 72 h, and the time was 6:00 UTC in the simulation. The simulation of the 1000 m-height backward trajectory can reveal the influence of long-distance transport of air masses on greenhouse gas concentrations at Akedala station.

### 2.3. Trajectory Clustering

Since the large number of trajectories generated by the air mass model are not suitable for quantification and visualization, cluster analysis was used to clarify the affinity of trajectories. The total spatial variance (TSV) is a method for determining the number of clusters [32]. The results of the cluster analysis based on the principles of TSV are highly representative and can reflect the differences in the heights and directions of the air masses. In this study, the backward trajectories arriving at the Akedala station from September 2009 to December 2019 were clustered (spring: March–May, summer: June–August, autumn: September–November, and winter: December–February of the following year). TrajStat software was used to process the simulated trajectories of each season, and the Euclidean distance was used to simulate the trajectories of air masses arriving at the Akedala station. The CO_2_ and CO concentration data were then mapped to the simulated trajectories to quantify the effects of air masses with different trajectories on CO_2_ and CO concentrations at the Akedala station.

## 3. Results and Discussion

### 3.1. Inter-Annual Variation in Atmospheric CO_2_ and CO Concentrations

**Figure 3:**
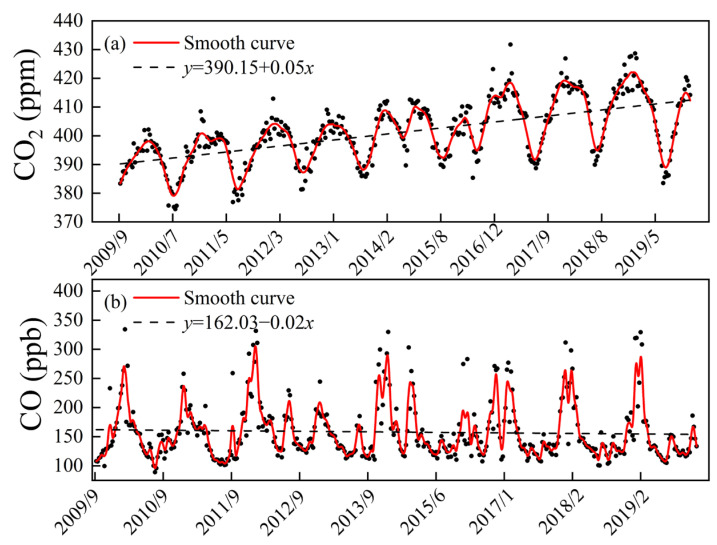
Inter-annual variation in CO_2_ (**a**) and CO (**b**) concentrations at Akedala station from September 2009 to December 2019. Notes: Black dots represent the observed concentrations, red solid lines are the fitted smooth curves for CO_2_ and CO concentrations, and dashed lines are the trend lines.

**Figure 4:**
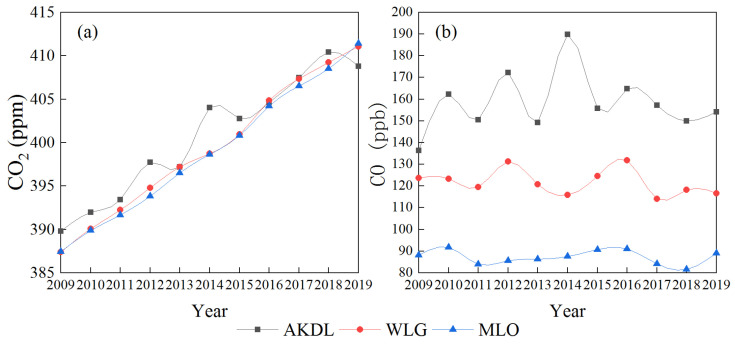
Comparison of annual average CO_2_ (**a**) and CO (**b**) concentrations at AKDL, WLG, and MLO stations from 2009 to 2019.

The annual average CO_2_ concentration at Akedala station showed a rising trend (ranging from 389.80 to 410.43 ppm) from September 2009 to December 2019, with an average annual growth rate of 1.90 ppm year^−1^, and the fitting equation was *y* = 162.03 − 0.02*x* (Figure 3a). The average annual CO_2_ concentrations at Waliguan and Mauna Loa stations during the same period were 399.45 ± 7.61 and 399.05 ± 7.60 ppm, respectively, and the average annual growth rates were 2.37 and 2.40 ppm year^−1^, respectively (Figure 4a). The average annual CO_2_ concentration at Akedala station (400.75 ± 6.78 ppm) was higher than that at Waliguan station and Mauna Loa stations over the 11 years. 

The annual average CO concentration at Akedala station between September 2009 and December 2019 ranged from 136.30 to 189.82 ppb, with an average annual concentration of 157.96 ± 13.38 ppb and an average annual growth rate of 1.78 ppb year^−1^, and the fitting equation was *y* = 390.15 + 0.05*x* (Figure 3b). The annual average CO concentrations at Waliguan and Mauna Loa stations were 121.80 ± 13.01 and 87.29 ± 3.09 ppb, respectively, and the average annual growth rates were 13.01 and 0.09 × 10^−9^ year^−1^, respectively (Figure 4b). The CO concentration at Akedala station showed a slightly decreasing trend, which was much higher than that of the other two stations. The fitting results of CO concentration at Akedala station showed that there was a slight downward trend, and the annual average concentration fluctuated greatly, with an amplitude of 35.95 ppb. At the same time, the CO concentration at Waliguan station showed a downward trend with an amplitude of 17.11 ppb, and the CO concentration at Mauna Loa station maintained a steady change with an amplitude of 9.37 ppb. 

**Figure 5:**
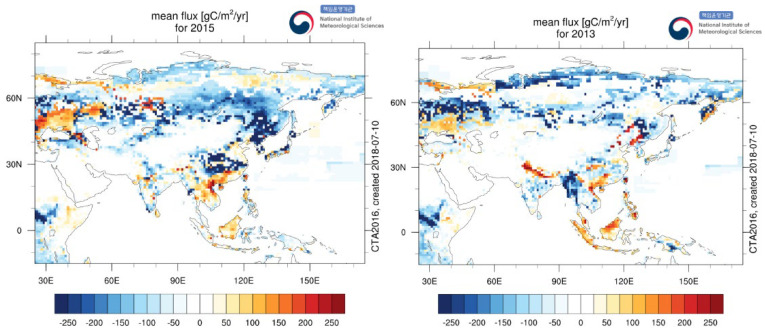
Flux Maps for 2013 and 2015 (This figure was downloaded from http://www.nims.go.kr/2/carbontracker/flux_maps.html, accessed on 19 May 2022). Note: The mean flux for the time period indicated. Negative fluxes (uptake) are displayed in blue colored pattern. Positive fluxes (emission) are red colored pattern. Units are gC/m^2^/year. The figures include biosphere and ocean fluxes. No fossil fuel and wildfire included.

The changes in the annual average concentrations of CO_2_ and CO were obviously different. During the 11 years, the concentration of CO_2_ increased obviously, while the concentration of CO showed a trend of first rising and then decreasing. The average annual growth rate of CO concentration was 10.70 ppb year^−1^ from 2010 to 2014, −7.15 ppb year^−1^ from 2015 to 2019, and 13.09 ppb year^−1^ from 2012 to 2014. This is due to the fact that the concentration of CO was affected by natural emissions, sinks, and meteorology. Studies have shown that the atmospheric self-purification capacity decreased from 2012 to 2014 [33]. This leads to the increase in CO concentration. It should be noted that the annual average concentrations of CO_2_ and CO decreased in 2013 and 2015. This is due to the following factors: First, CO is mainly from anthropogenic activities such as fossil fuel and biomass combustion. From 2012 to 2013, the proportion of coal-based energy consumption in Xinjiang dropped by 0.8%, while the proportion of clean energy consumption (solar energy, wind energy, etc.) increased by 1.3% between 2014 and 2015 [34]. As a result, the correlation between CO_2_ and CO sources changed [20], which proves that the anthropogenic emission of CO_2_ has a downward trend. Secondly, in 2013 and 2015, the average CO_2_ flux in Central Asia showed a strong uptake characteristic, the terrestrial carbon sink was stronger than in previous years, and the fossil fuel CO_2_ flux decreased (Figure 5). Thirdly, the analysis of ground-based meteorological data showed that the number of days with strong winds at the Akedala station in 2013 and 2015 was 43 and 39, respectively, which were higher than the annual average number of days with strong winds for the 11 years (34 days). Therefore, there were better diffusion conditions for CO_2_ and CO in 2013 and 2015. In summary, CO_2_ and CO concentrations are jointly affected by atmospheric self-purification capacity, terrestrial carbon sinks, meteorological factors, and energy consumption.

### 3.2. Seasonal Variation in Atmospheric CO_2_ and CO Concentrations

**Figure 6:**
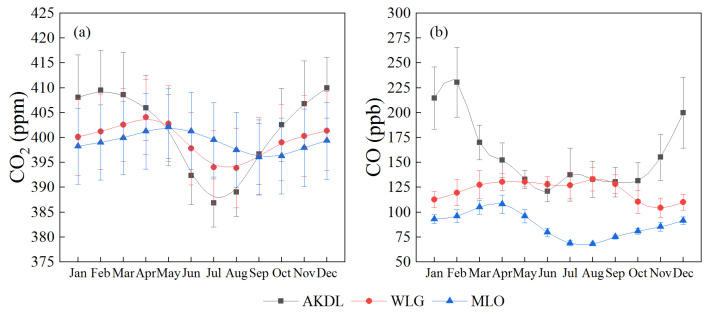
Seasonal variations in CO_2_ (**a**) and CO (**b**) concentrations at AKDL, WLG, and MLO stations. (Note: The bars are the overall standard deviation of the sample).

Seasonal changes were analyzed based on monthly average CO_2_ and CO concentrations at the Akedala station, which was then compared with seasonal changes at WLG and MLO stations. The maximum value of CO_2_ concentration (410 ± 6.11 ppm) at Akedala station appeared in December, and the minimum value (386 ± 4.84 ppm) appeared in July (Figure 6a). In spring and summer (from March to July), the CO_2_ concentration at the Akedala station showed a downward trend and remained at a low level in summer. The CO_2_ concentration increased from August to February of the following year. The monthly variation of CO_2_ concentration at the Akedala station was greater, with an amplitude of 24 ppm than at Waliguan station (10.25 ppm) and Mauna Loa station (10.01 ppm). The environment of the three stations varies greatly. There is a small amount of animal husbandry and human activities near Akedala station [35], and there is almost no animal husbandry and human activities near Waliguan and Mauna Loa stations [36]. Moreover, Akedala station has a temperate continental climate with obvious seasonal alternation, while Mauna Loa and Waliguan stations have a tropical marine climate and plateau continental climate, respectively, without obvious seasonal changes. This is the reason for the difference in the amplitude of monthly CO_2_ concentration change.

The CO_2_ concentration at Akedala station was the highest in winter (408.46 ± 7.48), followed by spring (405.66 ± 3.78 ppm), autumn (400.66 ± 8.00 ppm), and summer (390.47 ± 4.57 ppm). The growth rate was also the highest in winter (2.77 ppm year^−1^), followed by spring (2.05 ppm year^−1^), autumn (1.87 ppm year^−1^), and summer (1.19 ppm year^−1^). Therefore, the CO_2_ concentration and growth rate show a synchronous seasonal change and were higher in winter and spring. Altay, where Akedala station is located, has a long and cold winter, with an average temperature of −14.3 °C in winter and an average minimum temperature of −22.9 °C in winter. Moreover, there are many cold waves in spring. Due to the cold climate, Altay is heated a lot in winter and spring, so the CO_2_ concentration is higher in winter than in the other seasons. In summer, the sunshine hours can reach 325.2 h in June and 322.6 h in July. Suitable light and temperature conditions improve photosynthesis and absorption of CO_2_ by plants.

The CO concentration at Akedala station was highest in February (203.56 ± 35.09 ppb). It then declined rapidly until June (120.85 ± 10.43 ppb) and fluctuated at a low level until September (130.31 ± 10.01 ppb) (Figure 6b). After that, it increased rapidly until February of the next year. The peak CO concentration was 1.9 times higher than the lowest value. The monthly variation was significant. The annual average CO concentration was 157.96 ± 13.38 ppb. Only January, February, March, and December had CO concentrations above the annual average. The maximum CO concentration at Waliguan station was (130.51 ± 4.84 ppb) in May, and the minimum was (104.53 ± 9.62 ppb) in November. The maximum CO concentration at Mauna Loa station was (107.98 ± 9.26 ppb) in April, and the minimum was (67.99 ± 1.84 ppb) in August (Figure 5). The seasonal fluctuations of CO concentrations at WLG and MLO stations were smaller, and the CO concentration at WLG station was slightly higher than that at MLO station. Both stations are far from cities and less affected by anthropogenic activities. The difference in seasonal fluctuation is due to the difference in the photochemical reactions caused by natural conditions. MLO station is located at low altitude and is impacted by ocean currents, while WLG station is located at high altitude. The annual average temperature and humidity at MLO station were slightly higher than those at WLG station. This provides a good condition for the reaction of CO with OH [37]. 

The concentration of CO (215.78 ± 19.80 ppb) and growth rate (6.84 ppb year^−1^) at Akedala station were highest in winter (Spring: 154.16 ± 18.28 ppb, −3.62 ppb year^−1^; Summer: 131.48 ± 16.41 ppb, 0.93 ppb year^−1^; Autumn: 139.41 ± 13.55 ppb, −0.78 ppb year^−1^). This is consistent with the seasonal changes in atmospheric pollution [38]. CO is one of the air pollutants produced during fossil fuel and biomass combustion. Its concentration is closely related to anthropogenic emissions, air mass transport, radiation intensity, and the photochemical reaction of CO in the atmosphere. The low temperature and weak solar radiation in winter could suppress the photochemical reactions of CO and OH, leading to increased atmospheric CO concentrations [39]. The average temperature and average sunshine duration in winter at Akedala station were −14.3 ℃ and 5.46 h, respectively. Therefore, anthropogenic activities are the main driving factors for the high CO concentration in winter. In addition, high latitude, long and cold winters, cold Mongolian high pressure, temperature inversions for snow cover, and high atmosphere stability at Akedala station could also limit CO diffusion [38].

### 3.3. Correlation Analysis of Atmospheric CO_2_ and CO Concentrations

**Figure 7:**
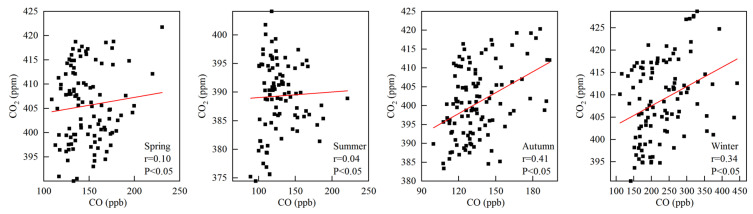
Correlation analysis of CO_2_ and CO concentrations in different seasons.

CO is not a greenhouse gas. It is produced during the incomplete combustion of fossil fuels and biomass. Therefore, it can be used as an indicator of anthropogenic pollution [40,41]. CO also plays an important role in atmospheric chemistry. The reaction of CO with OH removes about 75% of OH from the atmosphere (CO + OH→CO_2_ + H), which could affect the oxidative capacity of the atmosphere [6,42]. In this study, CO_2_ and CO concentration data of each sampling date were used in the correlation analysis, and the number of samples was 85 in spring, 74 in summer, 120 in autumn, and 110 in winter. The correlation between different seasons of CO_2_ and CO was analyzed (Figure 7). Our results showed that the concentration of CO_2_ was positively correlated with that of CO in spring (r = 0.1). This suggests that the sources of CO_2_ and CO are not consistent. This is due to the fact that in spring, the average temperature at Akedala Station is still low; therefore, anthropogenic activities such as heating are the main sources of CO_2_ and CO. However, in spring, the sunshine duration gradually increases, and the temperature rises, which promotes plant growth and increases the absorption of CO_2_ by plants. Furthermore, in spring, the concentration of O_3_ is high at Akedala Station, which could provide OH for CO photochemical reaction [35]. In summer, there was no obvious correlation between CO_2_ concentration and CO concentration (r = 0.04). This indicates the difference in the source between CO_2_ and CO. In summer, plant photosynthesis and soil respiration also increase with the increase in temperature [41], resulting in a decrease in atmospheric CO_2_ concentration. In addition, the temperature, humidity, etc., in summer could enhance the photochemical reaction of CO, resulting in a decrease in atmospheric CO concentration [20]. In autumn (r = 0.41) and winter (r = 0.34), the CO_2_ concentration was positively correlated with CO concentration. The main source of CO in autumn and winter is heating; therefore, the sources of CO_2_ and CO are basically the same. Biomass and coal combustion for heating usually starts in October, and the photochemical capacity decreases due to low temperatures, frequent cold airs, and snow-covered surface from October to February [20]. Therefore, anthropogenic activity is the main reason for the increase in CO_2_ concentration during this period. The correlation results also indicate that fossil fuel and biomass combustion are sources of CO_2_ in autumn and winter. 

### 3.4. Transport Pathway Analysis

**Figure 8:**
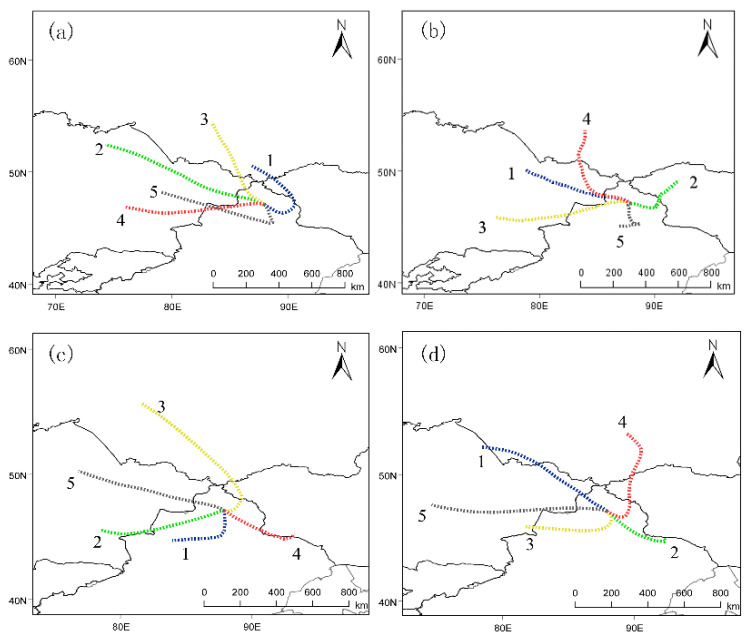
Clustering of backward trajectories at AKDL station in different seasons ((**a**), spring; (**b**), summer; (**c**), autumn; (**d**), winter).

**Figure 9:**
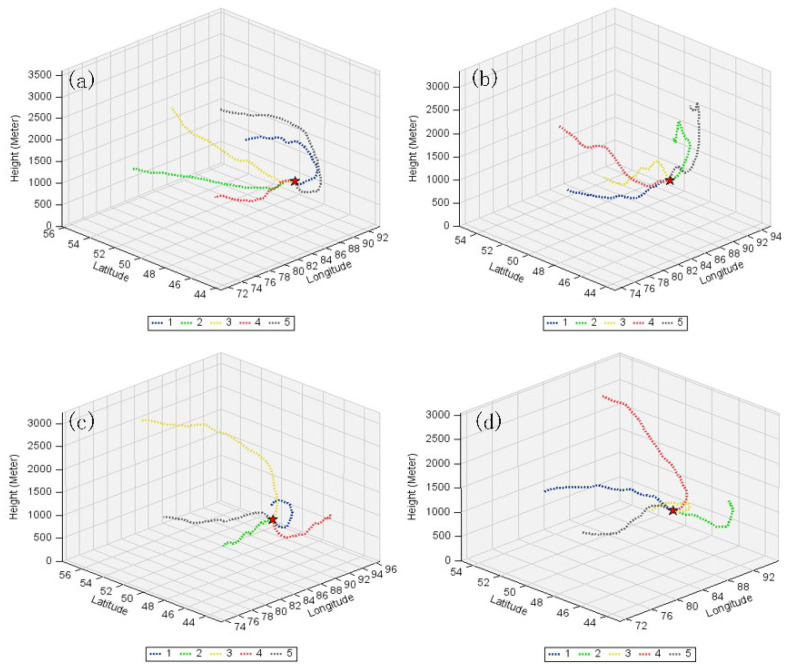
Three-dimensional height of backward trajectories at AKDL station in different seasons ((**a**), spring; (**b**), summer; (**c**), autumn; (**d**), winter).

The backward trajectories of air masses at Akedala station were clustered into five categories using the TrajStat, to determine the source areas and altitude of different trajectories (Figure 8 and Figure 9). The contributions of different trajectories to the CO_2_ and CO concentrations at the Akedala station were quantified (Table 1).

Akedala station is located in the west-northwest-trending plain between the Saur and Altay mountains [43]. The spring is characterized by cold weather with westerly and northwesterly winds. Our results showed that the air masses from eastern Kazakhstan accounted for 73.06% of the total at Akedala station and arrived along two trajectories (trajectories 1 and 4) from the northwest. Trajectories 1 and 4 were relatively low in CO_2_ and CO concentrations, with a CO_2_ concentration of 404.32 ± 3.01 and 403.40 ± 5.17 ppm, respectively, and a CO concentration of 152.65 ± 18.33 and 138.88 ± 13.14 ppb, respectively. The CO_2_ and CO concentrations of the air masses from eastern Kazakhstan were slightly lower than those of the air masses from southern Russia. The air masses from eastern Kazakhstan had a long trajectory and a fast-moving speed. This could facilitate the diffusion of pollutants and reduce CO_2_ and CO concentrations. The air masses from southern Russia accounted for 29.94% and arrived at Akedala station along three trajectories (trajectories 2, 3, and 5). Trajectory 2 moved along the northern foot of the Altai mountains and then crossed the Altai mountains from the east. Trajectory 3 directly reached Akedala station from the west of the Altai mountains through Habakhe. Trajectory 5 originated in southern Russia and passed through northwestern Mongolia. Trajectories 2, 3, and 5 had higher CO_2_ (405.20 ± 7.32, 408.27 ± 10.33, and 408.14 ± 7.86 ppm, respectively) and CO (148.26 ± 19.26, 161.53 ± 20.57, and 158.27 ± 19.57 ppb, respectively) concentrations. Therefore, CO_2_ and CO from southern Russia had a significant influence on CO_2_ and CO concentrations at Akedala station in spring.

In summer, strong convection provides good conditions for pollutant diffusion. Our results showed that the air masses from eastern Kazakhstan and southern Russia (via eastern Kazakhstan) accounted for 73.65%. Trajectories 1, 3, and 5 had a higher CO_2_ (388.89 ± 5.83, 389.77 ± 7.62, and 387.79 ± 5.05 ppm, respectively) and CO concentrations (134.92 ± 14.22, 139.51 ± 19.37, and 107.85 ± 12.79 ppb, respectively). The CO_2_ and CO concentrations of air masses of trajectories 1, 3, and 5 were higher than those of the other trajectories. Trajectory 5 moved downwards from high altitude, and trajectories 1 and 3 passed through Almaty and mining and industrially developed cities, reaching Akedala station from different directions. This could lead to increased CO_2_ and CO concentrations. Further, trajectory 3 passed through Karamay and other highly industrialized and densely populated cities, which could greatly increase the CO_2_ and CO concentrations at Akedala station. The air masses from southern Russia (via eastern Kazakhstan) accounted for about 25.26%. The CO_2_ concentration of trajectories 2 and 4 were 386.61 ± 7.07 ppm and 386.33 ± 1.93 ppm, respectively, and the CO concentration of trajectories 2 and 4 were 127.83 ± 13.18 and 138.65 ± 18.88 ppb, respectively. Most of the air masses arriving at Akedala station passed through eastern Kazakhstan; therefore, the CO_2_ and CO at Akedala station were mainly from eastern Kazakhstan in summer. However, in contrast to other seasons, the strong convection and better diffusion conditions in summer could lead to the reduction of CO_2_ and CO concentrations during long-distance transport [44].

In autumn, air masses from eastern Kazakhstan accounted for 58.68% of the total air masses arriving at Akedala station. Trajectories 2 and 5 originated in eastern Kazakhstan and passed through Karamay and Tacheng in China. Trajectories 2 and 5 had higher CO_2_ (403.27 ± 10.51 and 400.47 ± 8.60 ppm, respectively) and CO (139.16 ± 18.28 and 139.37 ± 17.55 ppb, respectively) concentrations. The proportion of air masses from northern Xinjiang and southern Altay Mountains increased. Trajectory 1 originated from the Xinjiang Tianshan North Slope Economic Belt and crossed the Junggar Basin, with CO_2_ and CO concentrations of 402.60 ± 8.76 ppm and 140.34 ± 16.71 ppb, respectively. This led to the CO_2_ and CO concentrations of trajectories 1, 2, and 5 being higher than those of other trajectories. The air masses from Russia’s southern Siberian region arrived at Akedala station from high-altitude pathways, while those of trajectories 3 and 4 were from relatively low-altitude pathways. The CO_2_ concentrations of trajectories 3 and 4 were 401.60 ± 8.03 and 391.31 ± 5.42 ppm, respectively, and the CO concentrations were 119.97 ± 13.72 and 116.71 ± 15.81 ppb, respectively. Trajectories 3 and 4 passed through sparsely populated areas with little anthropogenic activity and a clean atmosphere. As a result, the CO_2_ and CO concentrations after long-distance transport were lower than in other trajectories. Thus, in autumn, the main areas influencing CO_2_ and CO concentrations at Akedala station are eastern Kazakhstan and Northern Xinjiang.

In winter, the air masses from eastern Kazakhstan accounted for 39.79%, which was lower than in other seasons. The CO and CO_2_ concentrations of trajectories 4 and 5 were higher than those of other trajectories. The CO_2_ concentrations of trajectories 4 and 5 were 412.33 ± 9.00 and 409.58 ± 9.83 ppm, respectively, and the CO concentrations were 235.25 ± 24.75 and 228.19 ± 24.87 ppb, respectively. Trajectory 4 passed through large cities, including Semeyi, Burqin, and Habakhe in northeastern Kazakhstan, and trajectory 5 passed through Northeastern Kazakhstan, Karamay, and Tacheng.

The proportion of air masses from northern and southeastern Xinjiang increased in winter. The CO_2_ concentration of trajectories 1 and 2 were 412.05 ± 6.99 and 403.29 ± 8.05 ppm, respectively. The CO concentration of trajectories 1 and 2 were 227.93 ± 20.34 and 207.96 ± 18.33 ppb, respectively. The air masses of trajectory 1 originated in northern Xinjiang, and the CO_2_ and CO concentrations were higher in winter than in the other seasons. Under the influence of the Mongolian high pressure, the air masses of trajectory 1 moved rapidly. Mongolia, where much heating comes from coal and wood in winter, is the upstream area of the air masses, causing high CO_2_ and CO concentrations of trajectory 1. Trajectory 3 originated in southern Russia, with CO_2_ and CO concentrations of 408.02 ± 6.68 ppm and 219.16 ± 19.99 ppb, respectively.

In summary, eastern Kazakhstan, southern Russia, and Xinjiang Tianshan North Slope Economic Belt are the main source areas of CO_2_ and CO. The higher CO_2_ and CO concentrations in winter, when compared to the other seasons, is due to the air masses from the Xinjiang Tianshan North Slope Economic Zone of China, which carries more anthropogenic CO_2_ and CO. According to statistics, the gross production of energy and petrochemical industries in Northern Xinjiang accounts for 84% of the gross production of Xinjiang [45]. Furthermore, between 2009 and 2019, the territorial CO_2_ emission increased from 223.11 to 295.87 Gt CO_2_ in Kazakhstan, and the CO_2_ emission increased from 30.45 to 50.44 Gt CO_2_ [46]. This may be an important reason for the increase in CO_2_ concentration at the Akedala Station. Anthropogenic emissions and limited pollutant diffusion conditions are the main reasons for the higher CO_2_ and CO concentrations in winter.

## 4. Conclusions

In this paper, the CO_2_ and CO concentrations at the Akedala station between 2009 and 2019 were analyzed, and the inter-annual and seasonal variation characteristics, the correlation between CO_2_ and CO, and air mass transport pathways were also analyzed.

The annual average CO_2_ concentration ranged from 389.80 to 410.43 ppm between 2009 and 2019, with a growth rate of 1.90 ppm year^−1^. The annual average CO concentration ranged from 136.30 to 189.82 ppb, with a growth rate of 1.78 ppb year^−1^. Akedala station had a higher annual average concentration and growth rate than Waliguan and Mauna Loa stations. The concentration of CO_2_ and CO and growth rate were higher in winter than in other seasons. In addition, the concentration of CO_2_ was strongly correlated with CO concentration in autumn and winter, and fossil fuel and biomass combustion were the main sources of CO_2_. The correlation between CO_2_ and CO concentrations was weak, and the sources of CO_2_ and CO were different in spring and summer.

The main source areas of CO_2_ and CO were eastern Kazakhstan, southern Russia, and Xinjiang Tianshan North Slope Economic Belt of China. Most air masses passed through eastern Kazakhstan, with the main pathways parallel to the Erzis River valley and through the Old Wind Pass. The main reasons for the increase in CO_2_ and CO concentrations at Akedala station in winter are increases in easterly air masses from the northern slopes of the Tianshan Mountains and anthropogenic emissions. The limited conditions for pollutant diffusion during long-distance transport in winter also contribute to the high concentrations of CO_2_ and CO.

This study contributes to a better understanding of the long-term changes in CO_2_ and CO concentrations in Xinjiang, China (Central Asia). However, multi-site observations of CO_2_ and CO concentrations, as well as isotope analysis, are required to support the study on the sources of greenhouse gases.

## Figures and Tables

**Table 1:** Statistical results of CO_2_ and CO concentrations for back-trajectory clusters in different seasons.

Season	Clusters	Source Area of Air Masses	Percentage of Trajectories (%)	CO_2_ (ppm)	CO (ppb)
Spring	1	Eastern Kazakhstan, Hebukesai’er, Fuhai	34.03	404.32 ± 3.01	152.65 ± 18.33
	2	Southern Russia, Northeast Kazakhstan, Buerjin, Habahe	4.86	405.20 ± 7.32	148.26 ± 19.26
	3	Southern Russia, Altay, Beitun	18.19	408.27 ± 10.33	161.53 ± 20.57
	4	Southeastern Kazakhstan, Hebukesai’er	39.03	403.40 ± 5.17	138.88 ± 13.14
	5	Altai Mountain, Fuhai, Olgii, Fuyun, Qinghe	3.89	408.14 ± 7.86	158.27 ± 19.57
Summer	1	Southeastern Kazakhstan, Habahe, Buerjin, Beitun	27.15	388.89 ± 5.83	134.92 ± 14.22
	2	Southern Russia, Habahe	22.98	386.61 ± 7.07	127.83 ± 13.18
	3	Karamay, Alashankou, Tacheng	24.06	389.77 ± 7.62	139.51 ± 19.37
	4	Southern Russia, Northeast Kazakhstan, Buerjin, Habahe	2.28	386.33 ± 1.93	138.65 ± 18.88
	5	Northeastern Kazakhstan, Fuhai	23.52	387.79 ± 5.05	107.85 ± 12.79
Autumn	1	Southern part of the Junggar Basin], Tianshan North Slope Economic Belt, Fuhai	9.52	402.60 ± 8.76	140.34 ± 16.71
	2	Eastern Kazakhstan, Alashankou, Karamay, Tacheng	17.64	403.27 ± 10.51	139.16 ± 18.28
	3	Northern Altai Mountains, Southern Russia,	22.97	401.60 ± 8.03	119.97 ± 13.72
	4	Southern Russia, Altai Mountain, Qinghe, Fuyun, Fuhai	8.82	391.31 ± 5.42	116.71 ± 15.81
	5	Eastern Kazakhstan, Nur Sultan, Buerjin	41.04	400.47 ± 8.60	139.37 ± 17.55
Winter	1	Hovd, Olgii, Fuyun	34.41	412.05 ± 6.99	227.93 ± 20.34
	2	Tianshan North Slope Economic Belt, Northern part of the Junggar Basin	13.58	403.29 ± 8.05	207.96 ± 18,33
	3	Southern Russia, Altai Mountain, Qinghe, Fuyun, Fuhai	12.23	408.02 ± 6.68	219.16 ± 19.99
	4	Eastern Kazakhstan, Hebukesai’er, Fuhai	20.03	412.33 ± 9.00	235.25 ± 24.75
	5	Northeastern Kazakhstan, Karamay, Tacheng	19.76	409.58 ± 9.83	228.19 ± 24.87
